# Identification and characterization of aspartyl-tRNA synthetase inhibitors against *Mycobacterium tuberculosis* by an integrated whole-cell target-based approach

**DOI:** 10.1038/s41598-018-31157-3

**Published:** 2018-08-23

**Authors:** Ramón Soto, Esther Perez-Herran, Beatriz Rodriguez, Bogdan M. Duma, Monica Cacho-Izquierdo, Alfonso Mendoza-Losana, Joel Lelievre, David Barros Aguirre, Lluis Ballell, Liam R. Cox, Luke J. Alderwick, Gurdyal S. Besra

**Affiliations:** 1 School of Biosciences, University of Birmingham, Edgbaston, Birmingham B15 2TT UK; 2 Diseases of the Developing World, GlaxoSmithKline, Severo Ochoa 2, 28760 Tres Cantos, Madrid Spain; 3 School of Chemistry, University of Birmingham, Edgbaston, Birmingham UK

## Abstract

*Mycobacterium tuberculosis*, the causative agent of tuberculosis, has surpassed HIV as the leading cause of death due to an infectious disease worldwide, being responsible for more than 1.5 million deaths in low-income countries. In response to a pandemic threat by drug resistant strains, the tuberculosis research community is searching for new chemical entities with novel mechanisms of action to avoid drug resistance and shorten treatment regimens using combinatorial chemotherapy. Herein, we have identified several novel chemical scaffolds, GSK97C (spiro-oxazolidin-2-one), GSK93A (2-amino-1,3-thiazole, GSK85A and GSK92A (enamides), which target *M*. *tuberculosis* aspartyl-tRNA synthetase (Mt-AspRS), an essential component of the protein synthesis machinery of tuberculosi*s*, using a whole-cell target-based screening strategy against a genetically modified *Mycobacterium bovis* BCG strain. We also provide further evidence of protein inhibition and inhibitor profiling through a classical aminoacylation reaction and a tRNA-independent assay, respectively. Altogether, our results have identified a number of hit new molecules with novel mechanism of action for further development through medicinal chemistry as hits and leads.

## Introduction

Tuberculosis (TB) ranks first as the leading cause of death due to an infectious disease worldwide, overcoming HIV^1^. It represents a major threat to global health with one-third of the world’s population infected with latent tuberculosis, and multi-drug resistant (MDR) and extensively drug resistant (XDR) strains posing a significant potential threat to health care systems if left unaddressed. To effectively treat patients with MDR-TB, a 24-month-treatment regimen with second-line drugs, such as aminoglycosides and fluoroquinolones is needed, which unavoidably increases pill burden and potential side effects due to nephrotoxicity and damage to the central nervous system^2^. In order to achieve effective eradication of MDR and XDR-TB safer and more effective drugs are urgently needed with entirely novel mechanism of action^3^.

Phenotypic high-throughput screening (HTS) strategies against *Mycobacterium tuberculosis* have provided many promising new hits, representing a shifting strategy from classical target-based approaches^4^. Whole genome sequencing (WGS) of spontaneous resistant isolates generated against HTS hits *in vitro* has proven to be a valid initial starting point for target identification^5^. The discovery of TMC207^6,7^, now licensed as the FDA-approved drug bedaquiline^8^, was one of the first hits to be characterised using this approach of WGS of resistant isolates, highlighting the success of phenotypic screening campaigns^9^. However, further detailed biochemical and genetic evidence is required to elucidate the precise mode of action of small molecule hits as exemplified by the recent studies of inhibitors targeting MmpL3^10–12^.

Aminoacyl-tRNA synthetases have extensively been studied by many academic research groups to elucidate the kinetics of their two-step reaction mechanism^13,14^, their specificity towards their cognate amino acid and tRNA^15^ and their evolution^16^. Their utility as the target of anti-infective agents is demonstrated by the use of the clinically approved isoleucyl-tRNA inhibitor, pseudomonic acid A^17^, although drug discovery efforts against these targets has remained challenging due to: (I) the lack of translational whole-cell inhibitory activities, (II) off-target effects due to ATP competitiveness and (III) poor pharmacokinetic profiles^18^. A rhodanine compound was previously identified to target the aspartyl-tRNA synthetase of TB by WGS approaches^19^, which was then biochemically validated in a tRNA-independent assay^20^, encouraging further screening campaigns to find more potent and chemically tractable hits against this target. Herein, we have identified Mt-AspRS inhibitors by a whole-cell target-based screening of the so-called TB box^21^, a GSK library of 11,000 compounds (previously assessed against *M*. *tuberculosis H37Rv*) against the surrogate *Mycobacterium bovis* BCG strain genetically engineered to constitutively express the TB AspRS open-reading frame in a replicative pMV261 plasmid. Combining whole-cell and target-based screening methods allows the discovery of new chemical entities with potential to shorten early drug discovery programmes.

## Results and Discussion

### Identification of novel *M*.*tb* AspRS inhibitors by a whole-cell target-based screening assay

In this study we report the identification of a number of biochemically validated Mt-AspRS inhibitors identified using a target-based whole-cell screening assay in *Mycobacterium bovis* BCG genetically modified to constitutively express the Mt-AspRS open-reading frame. The GSK TB box compound collection of 11,000 compounds^21^ was used at three independent concentrations (0.5, 2.5 and 12 μM) and initial hits were confirmed based on inhibition shift between the two strains (calculated as % inhibition of *M*. *bovis* BCG pMV261 (empty plasmid) *minus* % inhibition of *M*. *bovis* BCG pMV261::Mt-AspRS [based upon duplicate data]) on ActivityBase (IDBS). Assay quality was monitored in an inter-plate manner with the statistical Z′, the gold standard to assess assay quality and reproducibility in HTS assays^22^. Plates with Z′ values below 0.4 were discarded for further analysis due to poor assay robustness.

**Figure 1:**
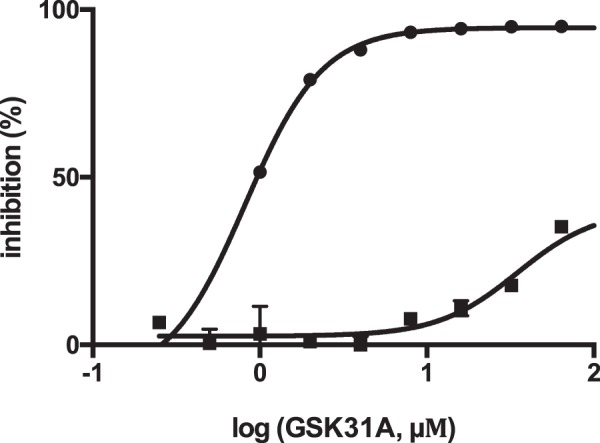
Dose-response *in vitro* activity curve of the tool rhodanine compound (GSK13A) showing target engagement between a pMV261::Mt-AspRS overexpressor *M*.*bovis* BCG strain (solid squares) and an empty pMV261 plasmid-containing *M*.*bovis* BCG strain (solid circles). Raw luminescence values from wells containing GSK13A (0.1–100 μM) were standardised to the positive (cells in 1% DMSO) and negative (7H9 media) control for cell growth. Processed data (percentage inhibition) was then plotted against each inhibitor concentration on Spotfire for MIC determination. Note the absence of inhibitory activities against the overexpressor strain at high compound concentrations, confirming resistance upon target overexpression.

Initial hits (250) were cherry-picked for further validation in a dose-response assay at a concentration range of 0.1 up to 100 μM to assess whole-cell potency and confirmation of whole-cell target-engagement (MIC50 shift) using the previously reported rhodanine entity as a tool control compound (Fig. 1). Compounds were tested in duplicate in an inter-plate manner and Sigmoidal dose-response curves were fitted to each data set using TIBCO Spotfire for analysis and data visualization. This resulted in the identification of 11 compounds with a minimum inhibitory concentration (MIC) shift >1. A table showing whole-cell target engagement is presented in the supplementary section (S1).

### Biochemical characterization of Mt-AspRS

**Figure 2:**
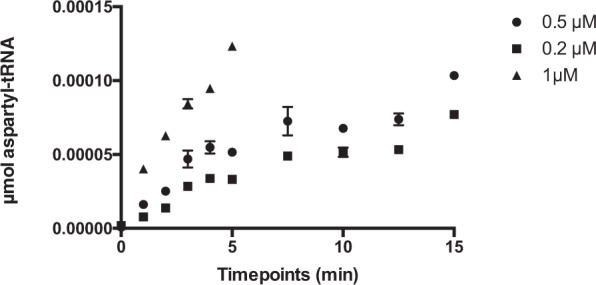
Time-course of the aminoacylation activity monitored using the standard TCA precipitation method at several Mt-AspRS concentrations. Background radioactive counts (corresponding to residual L-Asp in the absence of enzyme) were subtracted and data converted to amount of product (aspartyl-tRNA) over the time-course of the assay. The data was then plotted on GraphPad Prism 7.0 for the determination of the initial rate, which can be shown during the first 15 minutes of the aminoacylation reaction. 0.2 (triangles), 0.5 (circles) and 1 μM (inverted triangles).

**Figure 3:**
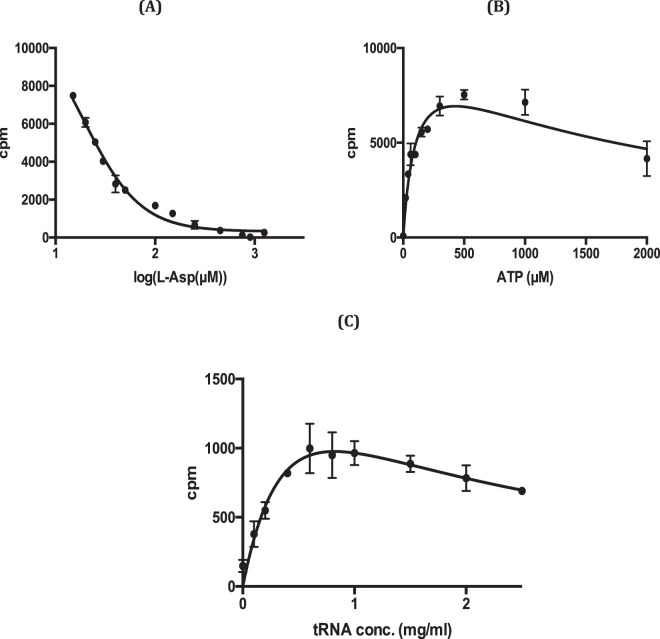
Substrate dependence of Mt-AspRS activity. **(A)** Sigmoidal dose-response curve was fitted to the isotopic dilution of L-Asp and K_M_ was determined as the IC50 minus the concentration of radiolabelled L-Asp. For **(B**,**C)**, hyperbolic Michaelis-Menten curves were fitted to obtain apparent K_M_ values for ATP and tRNA, respectively. Each experiment was done keeping two out of three substrates at saturation and K_M_ values are reported as the mean ± the standard deviation of two duplicates.

Due to potential pathway-related effects caused by target overexpression^23^, biochemical evidence of protein inhibition is essential to further validate on-target inhibitory activities against a given target. In order to mimic physiological conditions we have utilized a classical tRNA-based aminoacylation reaction with Mt-AspRS in a 96-well-format to assess inhibitor potency and guide future hit-to-lead medicinal chemistry programmes against this target. The assay length and enzyme concentration were adjusted in order to obtain an initial rate. The assay mixture was sampled at each time-point and precipitable radioactivity was quantified by scintillation counting. Background counts corresponding to free radiolabelled aspartic acid were subtracted at each data point and plotted over the time course of the assay (Fig. 2). For substrate dose-response studies, K_M_ values are shown in Table 1 obtained as the mean ± SD of two duplicate sets obtained from hyperbolic Michaelis-Menten curves fitted to data obtained from tRNA and ATP dose-response studies. L-Asp K_M_ values were obtained from the IC50 of a Sigmoidal curve minus the total concentration of radiolabelled aspartic acid employed in an isotopic dilution assay. Since a substrate with K_M_ in the low micromolar range do not contribute to the increase in enzyme velocity, the concentration of radiolabelled L-Asp was subtracted from the IC50 value of the Sigmoidal curve for an accurate determination (Fig. 3).

**Table 1:** Summary of the apparent K_M_ values for Mt-AspRS determined under Michaelis-Menten conditions.

Varied Substrate	Unvaried substrates	Km (app) (µM)
L-Asp	ATP (500 µM), tRNA (1 mg/ml)	13.8 ± 9.6
tRNA	ATP (500 µM), L-Asp (900 µM)	0.40 ± 0.22
ATP	tRNA (1 mg/ml), L-Asp (900 µM)	208.85 ± 8.41

Data is expressed as the mean ± standard deviation of two independent replicates and the correlation coefficient is provided as a measure of goodness of fit for each of the curves.

### Biochemical validation of screening hits against Mt-AspRS

**Figure 4:**
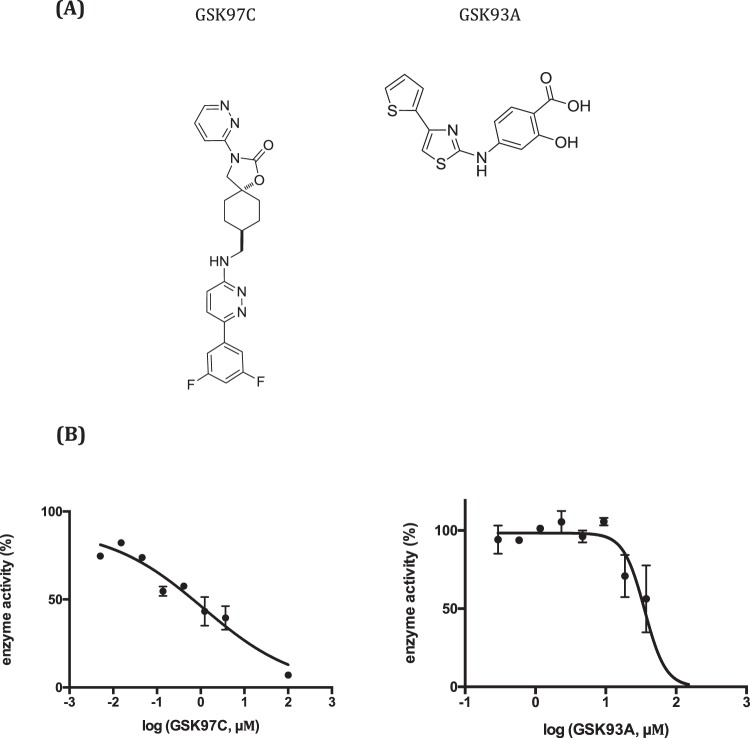
Structures and *in vitro* potency of novel antimycobacterial compounds that target the essential Mt-AspRS. **(A)** Structures of compounds identified through a target-based whole-cell screening assay against a *M*. *bovis* BCG over-expressing AspRS from *Mtb*. **(B)** Dose-response curves of the effect of compounds GSK97C and GSK93A against Mt-AspRS in the amino acylation reaction. Reaction mixtures were added to increasing compound concentrations (0.005–100 μM) and precipitable radioactivity quantified by scintillation counting in a Microbeta Trilux 1450. Raw cpm values were standardised to the maximum (100%) and minimum (0%) control for enzyme activity (with and without enzyme, respectively), plotted over the logarithm of inhibitor concentration and fitted to Sigmoidal dose-response curves for IC50 determination. Each inhibitor was tested in duplicates and the IC50 is reported as the mean of two replicates.

In this study two active novel chemical families (GSK93A and GSK97C, Fig. 4) previously identified in the target-based HTS assay using *M*. *bovis* BCG over-expressing AspRS from *M*.*tb* were validated when interrogated for potency against isolated enzyme in an aminoacylation reaction. Structural details and physicochemical and biological properties of these hits, including MIC values against BCG strains and HepG2 cell lines, biochemical IC50 and physicochemical data are briefly summarised in the supplementary section. *In vitro* biochemical dose-response validation assays were performed at 0.2 μM Mt-AspRS and matching apparent K_M_ values of L-Asp and tRNA while keeping ATP at a saturating concentration. Compound stocks were made as 10 mM solutions in 100% DMSO, diluted in a 1:3 fashion and dispensed into clear 96-well-polystetyrene plates up to a final concentration of 100 μM. The highest concentration of DMSO and CHAPS used in the assay were 1% and 0.5%, respectively, and precipitable radioactivity was transferred onto GF/C filter plates for scintillation counting. Raw data was standardized to the positive and negative enzyme activity controls and plotted versus the logarithm of the inhibitor concentration on Graph Pad Prism for IC50 determination.

### Medium throughput target-based screening assay against GSK97C, GSK87A and GSK93A analogues

**Figure 5:**
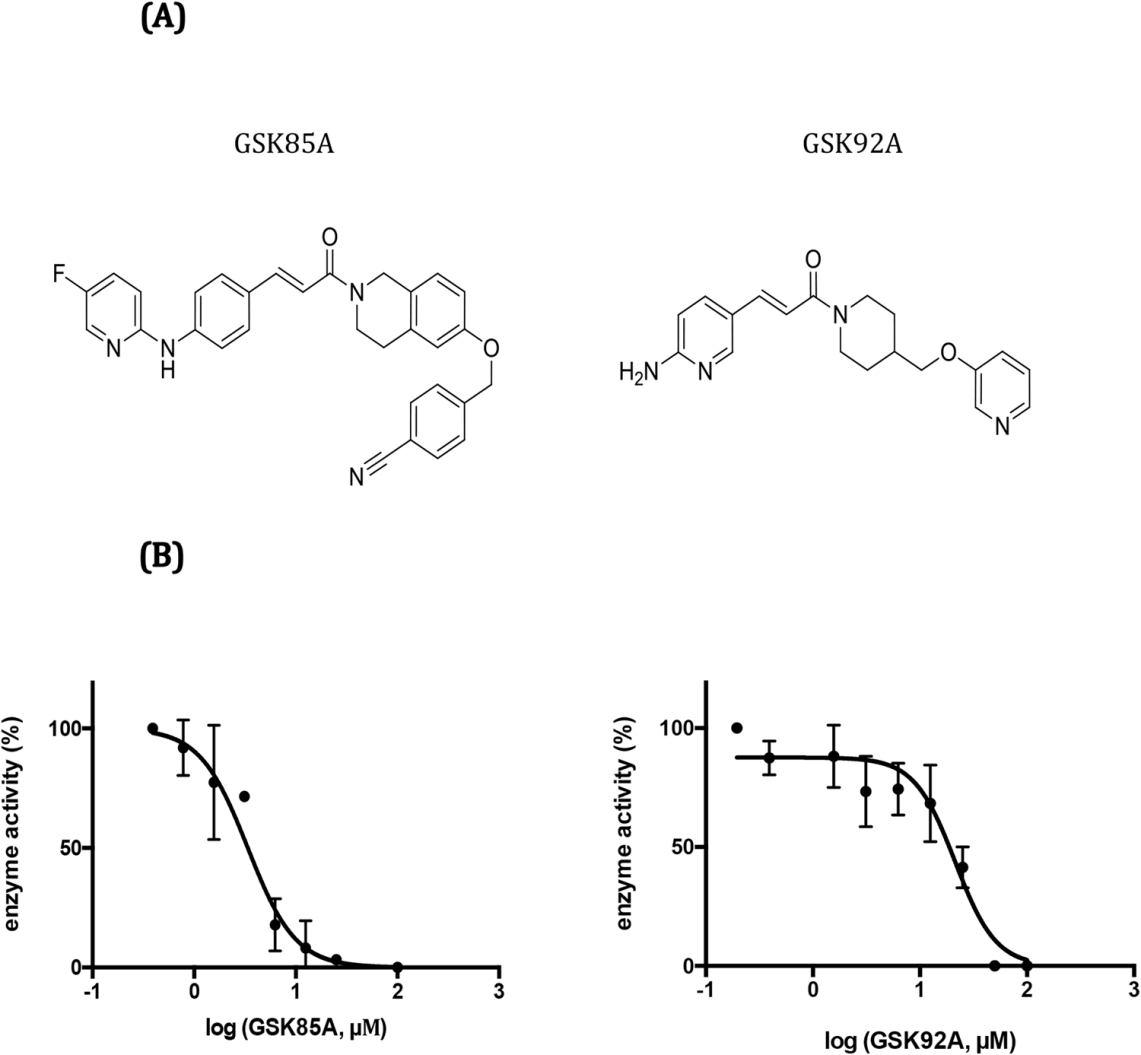
Structures and *in vitro* potency of newly identified antitubercular analogues of GSK97A targeting Mt-AspRS. **(A)** Structures of chemical entities identified in a target-based screening of structural analogues of several whole-cell screening hits in a tRNA-independent aminoacylation reaction. **(B)** Dose-response curves of the effect of compounds GSK92A and GSK85A against Mt-AspRS in the tRNA-independent amino acylation reaction. Raw fluorescence values corresponding to initial rates of the enzymatic reaction were normalised to the positive and negative control for enzyme activity (plus and minus enzyme, respectively) and plotted versus the logarithm of inhibitor concentration on GraphPad Prism 6.0 for IC50 determination.

As part of a preliminary approach to initial medicinal chemistry optimization efforts and therefore find chemical scaffolds with improved biological and physicochemical profiles, we performed a structure-based analogue search of these newly identified Mt-AspRS hits in the GSK 2 million compound collection to identify structurally-related hits that could be screened against Mt-AspRS in a target-based format. After attempts to miniaturise the tRNA-based assay into a 384-compatible SPA beads-based assay failed due to background inconsistency, we decided to employ a miniaturised tRNA independent assay optimised for HTS purposes. Surprisingly, two novel chemical entities, analogues of the inhibitor GSK87A previously identified in the whole-cell target-based AspRS overexpressor assay, were identified when a small library of analogues of the three validated hits were interrogated for their potency in the aminoacylation reaction at concentrations ranging from 0.005 up to 100 μM). Reagent stability was previously assessed over the overall period of the assay and robustness was monitored in an inter-plate manner (Z′ > 0.4). Interestingly, the parent compound GSK87A had failed to show potency in the biochemical validation assays against Mt-AspRS. However, two analogues of GSK87A (Fig. 5) were confirmed as Mt-AspRS inhibitors with IC50 values in the low micromolar range. The structures, biological and physicochemical properties are provided in the supplementary section (S2).

### Mechanistic characterization of novel Mt-AspRS inhibitors

**Figure 6:**
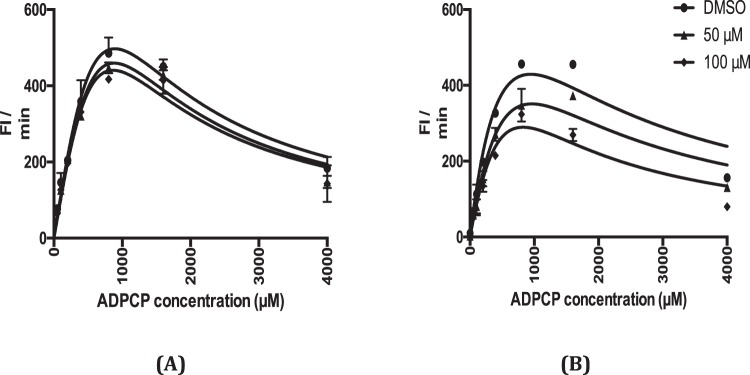
Reversible and non-competitive mode of inhibition of GSK97C **(A)** and GSK93A **(B)** against Mt-AspRS. β-γ-methyladenosine triphosphate (ADPCP, 0–4000 μM) and inhibitor (DMSO, 50 and 100 μM) were tested in duplicate in a microtiter 384-well-plate and the initial enzyme velocity (raw fluorescence units per minute) was plotted against each of the inhibitor concentrations to assess substrate competitiveness with regard to ADPCP.

Due to the importance to assess off-target inhibitory activities early on in drug discovery projects it is essential to gain mechanistic insights into compound binding, particularly when targeting proteins with universally conserved ATP binding sites, such as kinases^24^ or aminoacyl-tRNA synthetases^25^. Despite the apparent loss in potency observed for GSK97C (potentially due to compound instability in storage conditions) we show here how these inhibitors display non-competitive binding mechanism against Mt-AspRS with respect to a β-γ-methyladenosine triphosphate substrate as an ATP surrogate in a tRNA-independent reaction (Fig. 6), suggesting that the compounds interfere with tRNA aminoacylation in an allosteric-binding manner, away from the antiparallel beta-sheet ATP-binding pocket of class II aminoacyl tRNA synthetases.

## Conclusions

The availability of new chemical scaffolds with sufficient whole-cell and on-target inhibitory activities displaying novel modes of action represents a promising starting point in early TB drug discovery efforts. Traditionally, drug discovery efforts with aminoacyl tRNA inhibitors had been limited due (I) poor pharmacokinetic properties, (II) off-target effects caused by competitive binding mechanisms^18^ or (III) lack of penetration through bacterial membranes, the latter explaining the shifting strategy from target-based to whole-cell phenotypic approaches. We have expanded the chemical space available against Mt-AspRS that had traditionally been limited by these unspecific, ATP-competitive binding compounds or insufficient whole-cell inhibitory activities, further proving the versatility of Mt-AspRS as potential target for tuberculosis drug discovery. Diversifying the pipeline with inhibitors with novel modes of action is essential to ensure the overall survival of TB drug discovery projects with the potential to reach *in vivo* stages. Moreover, the success of integrating whole-cell and target-based screening strategies against other targets has previously been demonstrated, allowing the straightforward identification of small molecules against virtually any essential target with acceptable druggability features^26^. This has the potential to shorten the preclinical turnaround times of drug development projects. In this context, we have shown that expanding the chemical space available for Mt-AspRS inhibitors can expand existing possibilities for drug development by exploiting new hits with novel modes of action and mechanistic features, expanding the pipeline of available preclinical antitubercular agents. The identification of inhibitors targeting conserved binding sites that retain selectivity has been shown to be achievable^27^ although as we show here, mechanistic studies are needed to derisk potential ATP-competitive inhibitors. However, despite the availability of increasing numbers of preclinical candidates for the treatment of TB, it is important to consider that small molecule drug discovery is not enough to tackle the epidemic. Combinatorial chemotherapy needs to be complemented with rapid diagnostic tools and effective prophylaxis to contain the spread of drug resistant strains. In addition, improved health care is needed in low-income countries so that tuberculosis patients can effectively access new medicines. Altogether, our results represent another step toward the final eradication of TB by expanding the chemical space available against tRNA synthetases of this organism.

## Methods

### Ethics statement

All experiments were performed in accordance with the relevant ethical guidelines and regulations approved by the University of Birmingham and Diseases of the Developing World (DDW-GSK) Ethical Committees where required and there are no other ethical issues to report.

### Strains and culture conditions

Whole-cell screening experiments were conducted using *M*.*bovis* BCG that had been previously transformed with a pMV261 containing the Mt-AspRS^20^. Selected transformants were grown using 7H9 media supplemented with 0.025% Tween, 10% ADC (Albumin, Dextrose, Catalase) and kanamycin (25 μg/ml).

### Whole-cell target-based screening

The GSK TB box collection was screened for Mt-AspRS inhibitors based on resistance upon Mt-AspRS overexpression. We selected three different concentrations (0.5, 2.5 and 12.5 μM) based on compound potency in order not to miss any potential hit. A standard inoculum size of 10^4^ cells in 25 μL was added into each assay well of a 384-well-plate with white opaque walls using a *Multidrop*^*TM*^ *Combi* dispenser (Thermo Scientific). To control evaporation, plates were individually sealed with parafilm, covered with aluminium foil and stored in plastic boxes inside a 37 °C and 5% CO_2_ incubator with humidity control. At day 7, reconstituted Bac-titer GLO reagent (25 μL) were added into each assay well, which causes cell lysis and allows the luminescent detection of ATP consumption, briefly shaken for 8 minutes and read for luminescence using a Spectra Max M5 reader. Raw luminescence values were standardized to cell survival percentages using positive and negative controls for cell growth. The effect of a given inhibitor was calculated as: % inhibition = 100* ((data- control 1)/(control 2- control 1)) where control 1 = maximum activity (DMSO only; uninhibited growth), and control 2 = bacterial growth completely inhibited (by treatment with 10 μM rifampicin). Assay performance statistics (signal to background ratio and Z′) were calculated using templates in ActivityBase XE (ID Bussines Solutions Ltd, Surrey, UK). Dose-response confirmation studies were performed at a concentration range 0.1 up to 100 μM in a 1:3 dilution fashion and raw luminescence values were standardized to the maximum and minimum percentage of inhibition as stated for single-shot experiments. Dose-response curves were obtained on Tibco Spotfire and whole-cell target engagement confirmed by the MIC shift, reported here as the ratio between the mean of the MIC values for each strain.

### Biochemical characterization of recombinant Mt-AspRS

Pure, recombinant Mt-AspRS was obtained as previously described^20^. To establish accurate inhibitor potency, prior biochemical characterization is essential to obtain optimised substrate concentrations for aspartic acid (L-Asp), adenosine triphosphate (ATP) and tRNA. For speed and convenience, a pool of commercially available tRNA was used since a high degree of sequence identity is shared between *E*. *coli* and *M*. *tuberculosis* tRNA molecules. The Mt-AspRS functional characterisation was developed using a classical TCA precipitation method in standard 96-well-polystyrene plates. The aminoacylation reaction was performed in a final assay mixture of 80 μL consisting of 20 mM HEPES buffer pH 7.7, 4 mM MgCl_2_, 50 mM KCl and a range of substrate and enzyme concentrations in order to determine the initial rate and the kinetic constants of the aminoacylation reaction. The values obtained in the experiments described below were therefore obtained under steady-state conditions following classical Michaelis-Menten guidelines for enzyme kinetic studies. For the determination of the initial rate of the aminoacylation reaction, several enzyme concentrations (0.2, 0.5 and 1 μM) were incubated at 37 °C in the presence of 1 mg/mL tRNA from *E*. *coli* 600 MRE, 15 μM L-Asp (200 mCi/mmol, Perkin Elmer) and 4 mM ATP. The reaction was quenched at several time points over a period of fifteen minutes with 50 μL of 10% TCA. When the time-course was complete, the plates were incubated at 4 °C during 30 minutes to allow for proper tRNA precipitation. The aminoacylated product was then transferred onto GF/C 96-well filter plates (Packard), extensively washed in an excess volume of TCA (10%), once with 95% ethanol and then dried under a heat lamp prior to scintillation counting in a Microbeta Trilux 1450 model, basically in accordance with the method previously described for classical studies of aminoacyl-tRNA synthetases^28^. For K_M_ determination, varying substrate concentrations were assayed keeping the others at saturating concentrations. For L-Asp kinetic studies, a starter concentration of radiolabelled L-Asp of 15 μM was serially diluted with non-radioactive L-Asp to a final concentration of 900 μM where saturation was observed. The obtained data points were fitted to a Sigmoidal dose-response curve on Graph Pad Prism 7.0, and its IC50 value correSponds to the K_M_ plus the concentration of radiolabelled L-Asp used in the assay.

### Biochemical validation of screening hits

The IC50 dose response assays for each were performed at 0.2 μM Mt-AspRS and matching apparent K_M_ values of L-Asp and tRNA, while keeping ATP at a saturating concentration in order to filter out ATP-competitive inhibitors. Compound stocks were made as 10 mM solutions in 100% DMSO and dispensed into 96-well-polystetyrene plates in a 1:3 dilution manner up to a final concentration of 100 μM. In the maximum (100% activity) and minimum (0% activity) controls similar DMSO concentrations were added to check the absence of solvent-related inhibition. The highest concentration of DMSO and CHAPS used in the assay were 1% and 0.5%, respectively. Precipitable radioactivity was transferred onto GF/C filter plates for scintillation counting.

### Medium-throughput screening assay of a series of analogues in a tRNA-independent biochemical assay

A number of structurally related analogues to GSK97C, GSK93A and GSK87A from the GSK TB box collection were evaluated in a dose-response manner (0.1–100 μM) in a 384-well black-bottom microplate-adapted ATP release assay optimised for HTS purposes. The reaction mixture consisted of 20 mM HEPES pH 7.6, 4 mM MgCl_2_, 50 mM KCl, 1 mM DTT, 2 mM ADPCP, 10 mM D-glucose, 0.5 mM NADP^+^, 10 mM L- Asp, 3 μg of yeast hexokinase and *L*. *mesenteroides* glucose-6- phosphate dehydrogenase mixture and 0.5 μM of Mt-AspRS in a final assay volume of 20 μL. The amount of coupled enzymes used was high enough to minimise assay intereference. Stock compounds were dispensed with the Echo 555 (Labcyte) into black bottom 384 plates (Greiner 781101) and were stored at −80 °C until needed. Enzymes and substrates were kept separately in two working solutions prepared at 2X the final assay concentrations. Reactions were triggered with the addition of substrate solution into plates pre-plated with enzyme and no-enzyme control wells. The assay was performed at room temperature and fluorescence values were continuously monitored in EnVision (Perkin Elmer) during the period of the assay (4 minutes). Similar amounts of DMSO (1%) were added into control wells to check for absence of solvent-related inhibition. The raw data was normalised to the positive and negative controls for enzyme activity in Excel and was further processed in Excel and GraphPad Prism 6.0 for IC50 determination.

### Mechanistic characterization of novel Mt-AspRS inhibitors

The inhibitory activities of GSK97C and GSK93A against Mt-AspRS was assessed at several β-γ-methyladenosine triphosphate (0–8000 μM) and inhibitor concentrations (DMSO, 50 and 100 μM) while keeping fixed amounts of L-Asp and PPi at saturating values (10*K_M_). The reaction was triggered with the addition of 10 μL of a 2X substrate mix solution to a 384 black-bottom polystyrene plate (Corning) containing 10 μl of a 2X buffer solution with 1 μM Mt-AspRS in 40 mM HEPES pH 7.6, 8 mM MgCl_2_ and 100 mM KCl. Plates were then briefly centrifuged and read on EnVision (Perkin Elmer) using NADPH fluorescence in the kinetic mode as the method of detection. Slopes corresponding to initial reaction rates were plotted versus substrate concentration for each inhibitor concentration and data points were fitted to standard hyperbolic Michaelis Menten curves on GraphPad Prism 6.0 for K_M_ and V_MAX_ determination.

## Electronic supplementary material

Supplementary Information
