# Supplementary material for: Identification and characterization of aspartyl-tRNA synthetase inhibitors against Mycobacterium tuberculosis by an integrated whole-cell target-based approach
Source: Sci Rep. 2018 Aug 23;8:12664. doi: 10.1038/s41598-018-31157-3 (PMC6107548; doi:10.1038/s41598-018-31157-3)
Supplement: Supplementary file 1 — Supplementary Information [file 41598_2018_31157_MOESM1_ESM.pdf]

Supplementary Information

Identification and characterization of aspartyl-tRNA synthetase inhibitors against *Mycobacterium tuberculosis* by an integrated whole-cell target-based approach

Ramón Soto[1], Esther Perez-Herran[2], Beatriz Rodriguez[2], Bogdan M. Duma[3], Monica Cacho-Izquierdo[2], Alfonso Mendoza-Losana[2], Joel Lelievre[2], [2]David Barros Aguirre[2], Lluis Ballell[2], Liam R. Cox[3], Luke J. Alderwick[1] and Gurdyal S. Besra[1*]

[1] School of Biosciences, University of Birmingham, Edgbaston, Birmingham B15 2TT, UK

[2] Diseases of the Developing World, GlaxoSmithKline, Severo Ochoa 2, 28760 Tres Cantos, Madrid, Spain

[3] School of Chemistry, University of Birmingham, Edgbaston, Birmingham, UK

[*]Corresponding Authors: g.besra@bham.ac.uk

Table S1. Mt-AspRS target-engagement for several hits identified in a whole-cell target-based phenotypic screening assay. The MIC_{50} shift is calculated as the ratio between each compound MIC_{50} value (here shown as the mean of two inter-plate replicates per compound) against an Mt-AspRS *M.bovis* BCG overexpressor strain and an empty-plasmid control *M.bovis* BCG strain.

GSK ID	MIC *M.tuberculosis* H37Rv (µM)	MIC *M.bovis* BCG empty pMV261 (µM)	MIC *M.bovis* BCG pMV261::Mt-AspRS (µM)	Ratio (MIC shift)
GSK13A	2	0.7	6.7	9.6
GSK47B	17.5	4.9	32.8	6.8
GSK87A	0.9	0.8	5.1	6.4
GSK93A	31.25	0.4	1.7	4.3
GSK88A	17.8	0.5	1.6	3.2
GSK97C	20	10.5	24.9	2.4
GSK58A	9.3	7.2	15.3	2.1
GSK40A	10	11.7	18.9	1.6
GSK66A	> 80	1.8	4.7	2.6
GSK89A	5	25.4	50.1	1.9
GSK42A	> 80	8.8	10.5	1.19
GSK4A-V	80	1.7	3.0	1.7

Table S2. Biological and physicochemical characterization of several novel inhibitors targeting Mt-AspRS. Values are expressed as the mean of two independent replicates. ND: not-determined.

GSK ID	Structure	IC50 Mt-AspRS (µM)	MIC *M. bovis* empty pMV261 BCG (µM)	MIC *M. bovis* BCG pMV261::AspRS (µM)	MIC *M.tb* H37Rv (extra) (µM)	MIC *M.tb* H37Rv (intra) (µM)	HepG2 IC50 (µM)	CLND (µM)	clogP
GSK97C	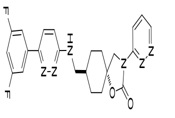	1.12	10.5	24.9	20	3.16	40	67	2.48
GSK93A	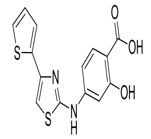	35.62	0.4	1.7	31.25	>50.11	>100	412	4.73
GSK92A	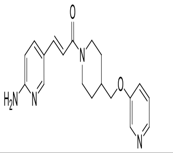	21.30	ND	ND	>80	>50.11	>100	276	1.43
GSK85A	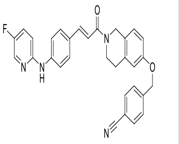	3.37	ND	ND	2.5	>50.11	25.11	4	6.10
